# Veterinary Department, Ohio State University

**Published:** 1896-10

**Authors:** 


### AMONG THE COLLEGES.

### VETERINARY DEPARTMENT, OHIO STATE UNIVERSITY.

THE year opened at this school with an increased number of students. Nine of the class will go up for graduation at the close of the college year. The department has been endowed by the board of trustees more liberally this year than heretofore, which enables the arrangement of a comfortable canine ward in the hospital building. The equipment of the hospital has been added to by many instruments, models for practical instruction, etc. The chair of anatomy and obstetrics will this year be filled by Dr. H. M. Ball. General and special surgery will be taught by Dr. W. F. Lavery. The subjects of theory and practice and veterinary medicine, horseshoeing, and meat-inspection by Dean Dr. D. S. White. The other branches in the several departments of the university.

The clinical facilities have been increased, and as a result better and more variety of patients. The past year, besides lamenesses, colics, pneumonias, etc., infectious and contagious diseases were represented. Among others, those of glanders, rabies, tuberculosis, equine chest-plague, cowpox, hog- and chicken-cholera, and a parasitic disease of sheep. Courses in practical operations upon narcotized animals and lectures upon milk- and meat-inspection have been added to the curriculum, both of which will tend to strengthen the course of instruction. The university is growing in appreciation by the people of the State.

